# Treinamento de anastomoses vasculares de baixo custo: o cirurgião vai à feira

**DOI:** 10.1590/1677-5449.000817

**Published:** 2017

**Authors:** Hícaro Donato Grahem, Renan Kleber Costa Teixeira, Daniel Haber Feijó, Vitor Nagai Yamaki, André Lopes Valente, Denilson José Silva Feitosa, José Maciel Calda dos Reis, Rui Sérgio Monteiro de Barros

**Affiliations:** 1 Universidade do Estado do Pará – UEPA, Belém, PA, Brasil.

**Keywords:** cirurgia, educação médica, capacitação, treinamento por simulação

## Abstract

Anastomoses vasculares são procedimentos comuns realizados por grande parte dos cirurgiões e cujo treinamento ocorre principalmente em seres humanos, contrariando os princípios éticos vigentes. Esse fato se deve, sobretudo, à carência e ao alto custo relacionados aos atuais modelos de treinamento. Assim, este estudo visa avaliar a viabilidade de três vegetais para a realização de anastomoses vasculares. Foram utilizadas cinco unidades de cebolinha, vagem e feijão-verde. Em cada uma tentou-se realizar uma anastomose término-terminal. Conseguiu-se a realização da anastomose apenas na vagem e no feijão-verde. Contudo, por apresentar uma menor espessura, o feijão-verde assemelhou-se mais aos vasos humanos.

## INTRODUÇÃO

A confecção de anastomoses vasculares é uma prática comum em diversas especialidades cirúrgicas, não estando restrita apenas aos cirurgiões vasculares e cardiovasculares, visto que em diversas situações há a necessidade de restabelecer o fluxo sanguíneo de um determinado órgão ou tecido, como, por exemplo, para a realização de retalhos, reimplantes e transplantes, e na cirurgia do trauma^1^^-^ 3.

Na maioria dos casos, o treinamento dessa habilidade é realizado pelos iniciantes da prática cirúrgica diretamente em seres humanos, podendo acarretar prejuízos ao paciente e aumento dos custos hospitalares por maior tempo de permanência e necessidade de procedimentos adicionais^2^^,^ 4 ^,^ 5. Na tentativa de minimizar esses danos, tem sido crescente o uso de simuladores na educação médica, pois possibilitam o treinamento em qualquer momento, reduzem os riscos para o paciente, proporcionam o controle do nível de dificuldade e permitem realizar treinamentos em etapas crescentes de habilidades^3^^,^ 6.

Assim, o treinamento prévio em simuladores deveria ser uma etapa fundamental na formação do futuro cirurgião^2^^,^ 3 ^,^ 6. Contudo, os elevados custos dos simuladores atuais disponíveis no mercado limitam a prática desse treinamento aos grandes centros. Nesse contexto, deve ser realizada a busca por modelos alternativos de baixo custo que consigam reproduzir o ganho de habilidades dos atuais sistemas, evitem o uso desnecessário de animais de experimentação e respeitem os atuais preceitos éticos^1^^-^ 7.

Diversos produtos de origem vegetal apresentam características semelhantes aos vasos sanguíneos (cilíndricos, longos e com uma luz interna). Assim, neste estudo buscou-se avaliar a viabilidade de três espécimes de vegetais como um modelo de baixo custo e fácil aquisição para a realização de anastomoses vasculares.

## MÉTODOS

**Figura 1:**
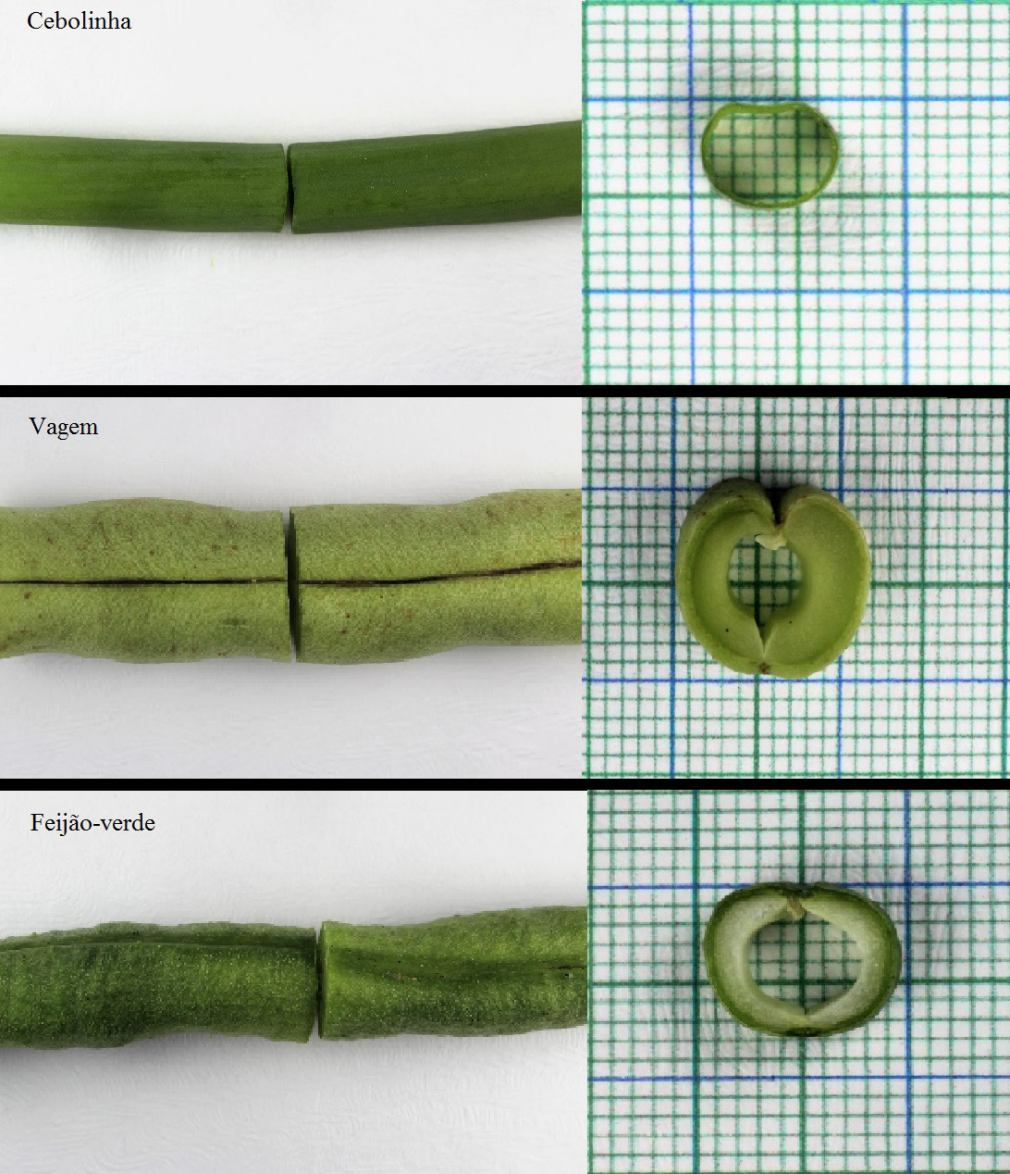
Características externas dos vegetais estudados.

Esse estudo caracteriza-se como experimental e avaliou a viabilidade de três vegetais para a realização de treinamento de anastomoses vasculares. Os modelos vegetais selecionados foram: 1) cebolinha (*Allium schoenoprasum*); 2) vagem (*Phaseolus vulgaris*); 3) feijão-verde (*Vigna unguiculata*) (Figura 1). Esses vegetais foram selecionados por apresentarem uma forma alongada e cilíndrica, além de apresentarem uma luz interna, assemelhando-se a vasos sanguíneos. Antes do início dos procedimentos, foram retiradas as sementes do feijão-verde, para garantir a patência inicial.

Foram testadas cinco unidades de cada espécime vegetal. Em cada uma foi realizada uma anastomose termino-terminal, por meio da técnica de triangulação^4^^,^ 6. Foi padronizada a realização de 10 pontos: dois nos ângulos de 0° e 180°, quatro na parede anterior e quatro na parede posterior dos vegetais. Foi utilizado o fio de polipropileno 6-0 para a realização dos pontos. Todos os procedimentos foram realizados por apenas um pesquisador.

Antes da realização das anastomoses, uma das extremidades do vegetal foi canulada com uma sonda vesical de alívio nº 16 conectada a uma seringa de 60 mL, e na outra foi realizado um furo na parede lateral com o auxílio de uma agulha 21G. Ambas as extremidades foram obliteradas com fio de algodão 0, permitindo assim a avaliação do fluxo inicial antes da realização do procedimento (buscando identificar possíveis soluções de continuidade) e após a confecção da anastomose; sendo esta considerada patente quando ocorreu a saída da água instilada pela cânula no orifício confeccionado.

Os parâmetros analisados foram custo do modelo, viabilidade para confecção da anastomose e patência antes da realização do procedimento e após a confecção da anastomose. Foram utilizados os *softwares* Microsoft® Word e Excel para análise dos dados e confecção dos gráficos e edição das fotos.

## RESULTADOS

**Figura 2:**
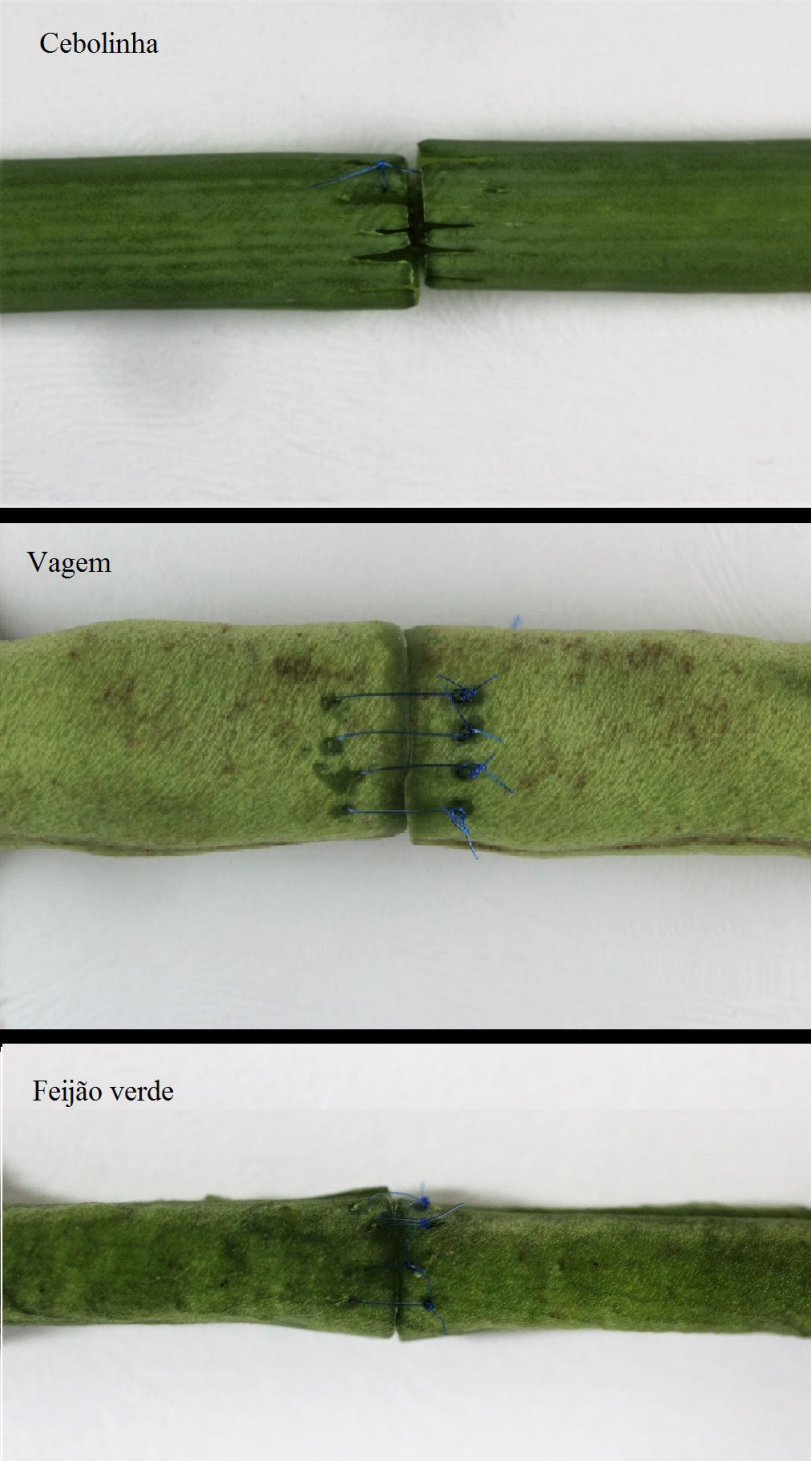
Aspecto final dos vegetais após a realização das anastomoses.

Todos os três vegetais testados apresentaram patência positiva antes da realização do procedimento. Em relação à confecção da anastomose, a cebolinha mostrou-se como um modelo inviável para a realização do treinamento, devido ao fato de suas fibras serem longitudinais e durante a confecção dos nós ocorrer o desprendimento destas, impedindo a correta técnica cirúrgica (Figura 2).

A vagem mostrou-se um modelo viável para a realização do treinamento de anastomoses, com patência positiva após o término do procedimento nos cinco espécimes testados. Porém, esse produto vegetal apresenta uma parede grossa, não simulando totalmente as características morfológicas de um vaso (Figura 1).

O feijão-verde, após a retirada de seus grãos, mostrou-se um modelo viável para a realização do treinamento vascular. Em todas as tentativas mostrou-se com uma patência final positiva; e as dimensões de suas paredes assemelham-se às das paredes dos vasos humanos. Os custos, comprimentos, diâmetros e espessuras das paredes de cada modelo de treinamento estão descritos na Tabela 1.

**Tabela 1:** Custos relacionados ao modelo de treinamento.

	**Preço**	**Quantidade**	**Comprimento**	**Diâmetro interno**	**Espessura da parede**
Cebolinha (*Allium schoenoprasum*)	R$ 1,60	45 unidades	27 cm	0,8 mm	1 mm
Vagem (*Phaseolus vulgaris*)	R$ 2,70	50 unidades	15 cm	1 cm	3 mm
Feijão-verde (*Vigna unguiculata*)	R$ 1,60	18 unidades	39 cm	1 cm	1 mm
Pinça anatômica	R$ 11,19	1 unidade	Não se aplica		
Porta-agulha	R$ 23,97	1 unidade	Não se aplica		
Tesoura	R$ 31,33	1 unidade	Não se aplica		
Caixa de luvas	R$ 22,00	1 caixa	Não se aplica		
Fio de polipropileno	R$ 35,00	6 caixas	Não se aplica		

## DISCUSSÃO

Anastomoses vasculares são procedimentos cirúrgicos básicos para diversas especialidades^1^^-^ 3. A curva de aprendizado dessa habilidade é longa, visto haver diversos fatores que modificam o desfecho final – a patência vascular. No início da aquisição dessa habilidade, a utilização de modelos alternativos não vivos que consigam simular as etapas cirúrgicas deve ser estimulada, a fim de minimizar os equívocos da prática em seres humanos^1^^,^ 3 ^,^ 4 ^,^ 8.

O presente estudo avaliou três modelos vegetais para a realização de anastomoses vasculares, visando gerar uma alternativa de baixo custo e facilmente reproduzível em diversos centros de treinamento. Entre os modelos estudados, apenas na vagem e no feijão verde foi possível realizar uma anastomose; sendo que o último apresentou uma vantagem, por conter uma menor espessura, simulando melhor as características morfológicas de um vaso arterial^9^. No entanto, o feijão-verde apresenta algumas limitações, como não conseguir simular uma importante característica morfológica das veias – a presença de válvulas^10^.

A cebolinha, por possuir suas fibras orientadas no sentido longitudinal, impediu a confecção da anastomose, porém sua parede muito delgada assemelha-se à das veias e, portanto, embora inviável para prática de sutura vascular, ao ser manuseada é capaz de simular a delicadeza das veias.

Devido à maior espessura da vagem em relação ao feijão-verde, esse modelo pode ser utilizado nas etapas mais iniciais, por manter melhor a conformação, facilitando a realização da anastomose^1^^,^ 3 ^,^ 5 ^,^ 8. Uma das desvantagens da utilização do feijão-verde é a retirada de seus grãos, que pode acabar por danificar o modelo de treinamento.

Outras modalidades de suturas vasculares, tais como longitudinal, oblíqua e dos tipos como término-terminal, término-lateral e látero-lateral, podem ser realizadas nesses modelos^2^^,^ 4 ^,^ 11. Fato relevante desse método é que os manejos e habilidades adquiridas podem ser aplicados a outras técnicas de suturas, como as anastomoses intestinais; contudo, dificilmente seria possível realizar os dois planos de sutura geralmente confeccionados nesse tipo de anastomose^12^.

Uma das limitações da utilização desses modelos vegetais é não poder ser avaliada de forma objetiva a qualidade da distância entre os pontos, pois, diferente do modelo *in vivo*, onde ocorre a coagulação entre os pontos, no modelo vegetal sempre haverá extravasamento entre os pontos, não permitindo assim saber se a distância entre os pontos foi adequada. Porém, essas características não inviabilizam a utilização desse modelo como etapa inicial de uma curva de aprendizado de diversos aspectos relacionados aos materiais, fios e tecidos utilizados^1^^-^ 5 ^,^ 8.

O custo para a realização do presente treinamento foi de aproximadamente R$ 320,00, sendo o maior custo relacionado à aquisição dos fios. Assim, acreditamos que a utilização de vegetais pode ser adotada para a prática de anastomoses vasculares, podendo ser inserida em programas de residência e graduação médica^12^. Porém, ainda há necessidade de melhoras no modelo estudado, como avaliações de anastomoses término-laterais, bem como a utilização desse sistema em manequins que simulem com maiores detalhes o procedimento cirúrgico e conectados a sistemas de pressão pulsátil^4^.

Assim, conclui-se que, entre as espécimes testadas, o feijão-verde apresentou as melhores características para ser utilizado no treinamento inicial de anastomoses vasculares, devido às suas dimensões e consistências semelhantes a vasos humanos e à sua resistência durante a confecção dos nós cirúrgicos.

## References

[1] Sigounas VY, Callas PW, Nicholas C (2012). Evaluation of simulation-based training model on vascular anastomotic skills for surgical residents. Simul Healthc.

[2] Kallás IE, Kallás AC, Callas E (1999). Anastomoses arteriais: passado, presente e futuro. Acta Cir Bras.

[3] Feliciano DV, Moore EE, Biffl WL (2015). Western trauma association critical decisions in trauma: management of abdominal vascular trauma. J Trauma Acute Care Surg.

[4] Eckstein HH, Schmidli J, Schumacher H (2013). Rationale, scope, and 20-year experience of vascular surgical training with lifelike pulsatile flow models. J Vasc Surg.

[5] Okhah Z, Morrissey P, Harrington DT, Cioffi WG, Charpentier KP (2013). Assessment of surgical residents in a vascular anastomosis laboratory. J Surg Res.

[6] Achar RA, Lozano PA, Achar BN, Pereira GV, Achar E (2011). Experimental model for learning in vascular surgery and microsurgery: esophagus and trachea of chicken. Acta Cir Bras.

[7] Brito CV, Soares RH, Botelho NM (2016). Laboratory animals and analgesia: the responsibility of ethics committees and the obligations of researchers. Rev Bioet.

[8] Amato AC, Freitas SL, Veloso PM, Correia TCV, Santos RV, Amato SJTA (2015). Gelatin model for training ultrasound-guided puncture. J Vasc Bras.

[9] Wilasrusmee C, Lertsithichai P, Kittur DS (2007). Vascular anastomosis model: relation between competency in a laboratory-based model and surgical competency. Eur J Vasc Endovasc Surg.

[10] Teixeira RK, Yamaki VN, Valente AL (2015). Existem válvulas na veia femoral em ratas Wistar?. J Vasc Bras.

[11] Price J, Naik V, Boodhwani M, Brandys T, Hendry P, Lam BK (2011). A randomized evaluation of simulation training on performance of vascular anastomosis on a high-fidelity in vivo model: the role of deliberate practice. J Thorac Cardiovasc Surg.

[12] Lima DA, Galvão MSL, Cardoso MM, Leal PRA (2012). Laboratory training program in microsurgery at the National Cancer Institute. Rev Bras Cir Plast.

