# Supplementary material for: Treinamento de anastomoses vasculares de baixo custo: o cirurgião vai à feira
Source: J Vasc Bras. 2017 Jul-Sep;16(3):262–6. [Article in Portuguese] doi: 10.1590/1677-5449.000817 (PMC5868946; doi:10.1590/1677-5449.000817)
Supplement: Translation

# Translation: Low-cost vascular anastomosis training: the surgeon goes to market

**Authors:** Hícaro Donato Grahem, Renan Kleber Costa Teixeira, Daniel Haber Feijó, Vitor Nagai Yamaki, André Lopes Valente, Denilson José Silva Feitosa, José Maciel Calda dos Reis, Rui Sérgio Monteiro de Barros

**Affiliations:** 1 Universidade do Estado do Pará – UEPA, Belém, PA, Brazil.

**Keywords:** surgery, medical education, training, training by simulation

## Abstract

Vascular anastomoses are common procedures and are performed by most surgeons. Training is primarily conducted in human beings, which violates current ethical principles. This is because current training models are expensive and in short supply. This study was designed to investigate the feasibility of three vegetable models of vascular anastomosis. Five units each of scallions, green beans, and yardlong beans were used. An end-to-end anastomosis was attempted with each specimen. Anastomoses were only successful in green beans and yardlong beans. Since they are narrower, the yardlong beans are the most similar to human vessels.

## INTRODUCTION

Constructing vascular anastomoses is a common practice in many surgical specialties and is not limited to vascular and cardiovascular surgeons, since there are many situations in which it is necessary to reestablish blood flow to an organ or tissue, such as, for example, for patches, reimplants, and transplants, and in trauma surgery.^1^^-^ 3

In the majority of cases, surgery novices’ first training in this skill is given in human beings, which can result in harm to patients and increased hospital expenses because of increased length of stay and additional procedures.^2^^,^ 4 ^,^ 5 With the objective of reducing this harm, there has been increasing use of simulators in medical education, because they enable training at any time, reduce the risk to patients, allow the level of difficulty to be controlled, and allow training to be conducted at successively higher skill levels.^3^^,^ 6

Therefore, preliminary training with simulators should be a basic step in the education of future surgeons.^2^^,^ 3 ^,^ 6 However, the elevated costs of the simulators currently available on the market have restricted this type of training to large centers. There is therefore a need to identify alternative models that are less expensive, that can reproduce the skill acquisition offered by current systems, that avoid unnecessary use of animals in experiments, and that respect current ethical principles.^1^^-^ 7

Many products of vegetable origin have similar characteristics to blood vessels (long, cylindrical, and with an internal lumen). This study was conducted to assess the feasibility of three types of vegetables for use a as a low-cost and easily-obtained model of vascular anastomosis.

## METHODS

This is an experimental study to evaluate the feasibility of using three types of vegetable for training in vascular anastomoses. The vegetable models chosen for the experiment were: 1) scallions (*Allium schoenoprasum*); 2) green beans (*Phaseolus vulgaris*); 3) and yardlong beans (*Vigna unguiculata*) (Figure 1). These vegetables were chosen because they are long and cylindrical in shape and have an internal lumen, similar to blood vessels. Before starting the procedures, seeds were removed from the yardlong beans, to achieve initial patency.

**Figure 1:** External characteristics of the vegetables studied.

Five units of each vegetable specimen were tested. In each case an end-to-end anastomosis was attempted, using the triangulation technique.^4^^,^ 6 The method was standardized with 10 stitches: two at angles of 0° and 180°, four in the anterior wall and four in the posterior wall. Sutures were performed using 6-0 polypropylene thread. All procedures were performed by a single researcher.

Before performing each anastomosis, one of the extremities of the vegetable specimen was cannulated with a nº16 urinary catheter connected to a 60 mL syringe, and at the other extremity a hole was bored in the side wall using a 21G needle. Both extremities were ligated with 0 cotton thread, enabling assessment of initial flow before conducting the procedure (attempting to identify possible sources of leakage) and after anastomosis; which was defined as patent if the water instilled via the cannula exited via the lateral orifice.

The parameters analyzed were cost of the model, feasibility of anastomosis and patency before the procedure and after anastomosis. Microsoft® Word and Excel were used for data analysis, to plot graphs, and to edit photographs.

## RESULTS

All three vegetables tested exhibited positive patency before the procedures. With relation to construction of anastomoses, it did not prove to be feasible to use scallions as a model for training because they have longitudinal fibers and knots came loose while they were being tied, making it impossible to accomplish the correct surgical technique (Figure 2).

**Figure 2:** Final appearance of the vegetables after anastomoses.

Green beans proved to be feasible as a model for training anastomoses, with positive patency after the procedure in all five specimens tested. However, this vegetable product has a thick wall and does not fully simulate the morphological characteristics of a human vessel (Figure 1).

After the seeds were removed from the yardlong beans, the empty pods proved to be a feasible model for vascular training. All attempts resulted in positive final patency; and the dimensions of their walls are similar to those of the walls of human vessels. The cost, length, diameter, and wall thickness for each training model tested are listed in Table 1.

**Table 1:** Costs related to training models.

	**Price**	**Quantity**	**Length**	**Internal diameter**	**Wall thickness**
Scallions (*Allium schoenoprasum*)	R$ 1.60	45 units	27 cm	0.8 mm	1 mm
Green beans (*Phaseolus vulgaris*)	R$ 2.70	50 units	15 cm	1 cm	3 mm
Yardlong beans (*Vigna unguiculata*)	R$ 1.60	18 units	39 cm	1 cm	1 mm
Anatomic forceps	R$ 11.19	1 unit	Not applicable		
Needle holder	R$ 23.97	1 unit	Not applicable		
Scissors	R$ 31.33	1 unit	Not applicable		
Box of gloves	R$ 22.00	1 box	Not applicable		
Polypropylene thread	R$ 35.00	6 boxes	Not applicable		

## DISCUSSION

Vascular anastomoses are essential surgical procedures in many different specialties.^1^^-^ 3 The learning curve for acquiring this skill is long, because there are a large number of factors that modify the final outcome of vascular patency. During the early stages of acquisition of this skill, use of alternative, non-living, models that can be used to simulate surgical steps should be encouraged, thereby minimizing mistakes in human beings.^1^^,^ 3 ^,^ 4 ^,^ 8

This study evaluated three vegetable models of vascular anastomoses, hoping to identify a low cost alternative that could be easily reproduced at different training centers. Of the models studied, it was only possible to achieve successful anastomosis in the green beans and yardlong beans; the second of which appears more advantageous because it is less thick and better simulates the morphological characteristics of an arterial vessel.^9^ However, there are also limitations to the utility of yardlong beans, primarily the fact that they cannot be used to simulate an important morphological characteristic of veins – their valves.^10^

Since scallions have fibers aligned longitudinally, it was not possible to construct an anastomosis with them, but their very fine walls are similar to veins and although they cannot be used to practice vascular sutures, when handled they do simulate the delicacy of veins.

The greater thickness of green beans in comparison with yardlong beans make this model suitable for the initial stages of training, since they maintain their shape better, facilitating anastomosis.^1^^,^ 3 ^,^ 5 ^,^ 8 One of the disadvantages of using yardlong beans is the need to remove the beans from the pods, which could damage them as a training model.

Other types of vascular sutures, such as longitudinal, oblique and types such as end-to-end, end-to-side, and side-to-side, can also be conducted with these models.^2^^,^ 4 ^,^ 11 Another relevant fact related to this method is that the handling techniques and skills acquired can be applied to other suture techniques, such as intestinal anastomoses; although it is unlikely to be possible to achieve the two suture layers generally used in this type of anastomosis.^12^

One of the limitations of using these vegetable models is that an objective assessment cannot be made of the quality of the distance between stitches, since, in contrast with in vivo models in which coagulation occurs between sutures, in the vegetable model there will always be leakage between sutures, so this cannot be used to determine whether the distance between stitches is correct. Nevertheless, these characteristics do not rule out using this model as an initial step in the learning curve of several aspects related to materials, threads and tissues.^1^^-^ 5 ^,^ 8

The cost of the training described was approximately R$320.00, with the greatest cost related to acquisition of suture threads. We therefore believe that use of vegetables could be adopted for practicing vascular anastomoses and could be incorporated into residency and undergraduate medicine programs.^12^ However, there are several improvements that need to be made to the model, such as assessing side-to-end anastomoses, and using it in dolls that simulate surgical procedures in greater detail and are connected to pulsating pressure systems.^4^

It is therefore concluded that, of the models tested, yardlong beans exhibited the best characteristics for use in initial training in vascular anastomoses, because it has dimensions and consistency similar to human vessels and because of its resistance when tying surgical knots.
